# Analysis of Two-Piano Teaching Assistant Training Based on Neural Network Model Sound Sequence Recognition

**DOI:** 10.1155/2022/5768291

**Published:** 2022-06-02

**Authors:** Lei Dai

**Affiliations:** Department of Art, Hefei Preschool Education College, Hefei, Anhui 230013, China

## Abstract

In today's society, with the gradual improve5ment of material living standards, people are also more in pursuit of their own spiritual enjoyment. The study of piano has gradually become a way for people to enrich their spiritual life, and more and more people attach importance to it. In the field of piano teaching, the two-piano method is a unique form of playing the piano. In order to solve the problem that the recognition accuracy of the sequence of two pianos is seriously reduced in the environment of noise and reverberation, this paper proposes an auxiliary training analysis system based on the neural network model. Firstly, in order to learn the nonlinear relationship between the sound order and the target task label from the massive data, a multitask preprocessing method combining speech enhancement and detection is used to supervise the deep neural network training. Then, convolutional neural network is used to construct the end-to-end recognition system, and the initial recognition results are checked and corrected by the phonological sequence model. Finally, the sequence recognition is carried out under the condition of noise, and the articulation is improved by speech enhancement front-end module, and then the sequence recognition model is used for recognition. Compared with traditional training methods, it is proved that our method is effective in improving the training efficiency and performance quality of players. At the same time, this method breaks through the limitation of traditional training method of double piano, creates a more scientific training means, and realizes the practice and application of artificial intelligence technology in the teaching of double piano.

## 1. Introduction

Compared with foreign countries, the systematic implementation of two-piano teaching in China started late. As early as the twentieth century, systematic teaching of double piano has been carried out abroad, and it has been widely applied in the music teaching courses of various colleges and universities. In recent years, China gradually began to attach importance to this scientific and efficient teaching method and began to apply it in universities. But, even so, the two-piano teaching method in China is only in the preliminary exploratory stage [1]. In general, some schools in China do not pay special attention to the two-piano teaching method, and only a small number of teachers have conducted in-depth research on this method, so it is difficult to popularize the two-piano teaching method, and then the development of this teaching method has a great resistance.

At present, many universities in China have piano majors, but compared to general piano teaching, two-piano teaching has not yet formed a perfect set of practical teaching methods, and it is not possible to maximize the use of the school's teaching resources and equipment. Therefore, it is necessary to further improve the construction of two-piano teaching staff. There are many colleges and universities that open piano classes, because the piano teaching conditions are relatively backward, so the number and quality of piano rooms and pianos cannot fully meet the two-way requirements of teachers and students for double piano teaching. In addition, due to the gradual improvement of people's living standards, Chinese parents pay more and more attention to the cultivation of children's artistic quality, and piano practice has gradually become the first choice of many families for the cultivation of children's quality [2]. The initiative and enthusiasm of Chinese teenagers towards piano learning are increasing year by year, followed by the opening and gradually attaching importance to piano-related majors in all kinds of music colleges. In general, the reason is that most schools offering piano-related professional courses have not timely formed a set of specific plans for two-piano teaching practice which are suitable for the students of this major and the actual teaching situation of their own schools [3]. Obviously, this negative attitude will to a large extent hinder the large-scale promotion and rational application of two-piano teaching method in China, which is the manifestation of the lack of responsibility as an educator and also a test for the construction of school infrastructure and allocation of education and teaching resources.

Music and computer are two completely different fields, and they have essential differences; however, with the continuous development of the times, this difference is gradually narrowing, and they form a complementary relationship. As a result of the constantly updated computer technology, and the voice of piano teaching reform, driven by the wave, the application of computer technology in piano teaching can be realized, and research on computer-aided piano teaching and works also have emerged, such as the Cui “the application of multimedia teaching means in piano teaching.” This gave the piano teaching of the overall level of a greater improvement [4]. With the rapid development and update of computer technology, sequence software has become a teaching method to improve the quality of teaching with its powerful function and simple humanized interface management.

In the past decade, the rapid development of deep learning technology has led to a technological revolution in the field of sequence recognition, and the recognition ability of machine systems under ideal conditions has been greatly improved. Many advanced technologies have a recognition rate of more than 95 percent. Under the background of this technology, intelligent products related to the analysis of piano teaching aid quickly open the market. Compared with the deep learning neural network model, more parameters need to be set. Min proposed a nonspecific isolated sequence recognition system based on the dynamic time regularization algorithm. The system could not carry out real-time sequence recognition and did not reflect the convenience of real-time detection in traditional methods. Liu used the classification and recognition method based on semisupervised learning to make an overall design of the sequence recognition system, but the system mainly aimed at the sequence recognition for specific sounds, which was not conducive to the promotion of the sequence recognition system. Dai et al. used feedforward sequence memory neural network to conduct acoustic modeling and sequence modeling for sequence recognition, which significantly improved the performance and training efficiency of the sequence recognition system. However, the performance of the sequence recognition system was still not ideal in the case of strong noise and far field. However, the model only underwent training and testing with a small amount of data, and there are still many theoretical and application problems to be further studied. Yang improved the convolutional neural network algorithm and adopted a new log activation function, which effectively improved the performance of CNN's sequence recognition and alleviated the overfitting problem in sequence recognition. However, at present, one of the problems restricting intelligent sequence recognition is that it is still difficult to improve the recognition rate of intelligent sequence recognition in complex situations such as noise and reverberation [5]. The human auditory system can accurately find the corresponding sequence in the complex environment full of various noises and interference and can recognize the sequence well, but the machine system cannot. Therefore, this paper takes robust sequence recognition methods as the research object, explores preprocessing methods and models that can improve sequence recognition, and applies them to the analysis of two-piano teaching auxiliary training, aiming to help players improve their playing skills without manual teaching.

## 2. Related Works

### 2.1. Two-Piano Teaching Status

In this traditional teaching process, teachers confirm whether there are any mistakes in students' learning and playing by listening to the tunes played by students and observing whether their fingering is correct. The traditional teaching mode and teaching ideology tend to make students feel uneasy in the learning process, resulting in students not being able to devote themselves to learning, and this will cause students to have fear of learning in their hearts for a long time, and even develop an aversion to learning. Double-piano teaching method has more strict requirements for the teachers and students; it requires precise cooperation between teachers and students; the traditional single-play mode escalated into a double-play one; the method of mutually beneficial cooperation can not only for promote communication between teachers and students but also ease the boring classroom atmosphere. Therefore, it can improve students' learning enthusiasm and learning effect. As a musical instrument with beautiful sound, the piano can make people feel rich and aesthetic experience in hearing, while the double-piano performance method can make up for the singleness of single piano to a certain extent, so as to make people's hearing experience fuller and richer. When teachers and students play together, students can better understand the overall music and also the value of students' interest can be improved [2]. This teaching mode greatly strengthens the overall ability of students to learn music. To sum up, the two-piano teaching method is of great significance to the cultivation of students' musical thinking and musical aesthetics.

At the end of the twentieth century, two-piano performance gradually appeared in China's performance stage, and its unique performance form and novel music style were favored by music lovers in China. In the field of piano education in China, double-piano teaching is gradually developed. Double-piano performance belongs to the category of chamber music ensemble. Both players should not only complete their own voice parts independently but also take into account the mutual cooperation, which is the perfect unity of generality and personality. In the learning process, the performance of individual skills and the expression of emotions are closely related. In the performance, it is required not only to have harmony and unity but also to highlight individual personalities and unique ways of music processing and expression. Many piano soloists have long been accustomed to practicing alone and thinking for themselves. There will be various problems in the training of two pianos, but the key is to find out whether these problems are caused by playing skills or musical understanding [6]. If the cause of the problem is not clear, the training process of two pianos will become an inefficient “training partner” process, and this kind of repeated training will not achieve a good performance effect.

The traditional teaching methods of two pianos are as follows: The first method is study score, where players can grasp the overall structure and ideological connotation of music works through joint study of score. The second method is using auditory sense to mobilize sensitive auditory sense. When performing, we should not only listen to our own performance but also listen to the performance of the partners and adjust our performance by listening to the changes and processing of the partners. The third method is a lot of practice, first of all, to achieve mastery of fingering, playing, phrase, rhythm, speed, structure, and paragraph cohesion of the music after practice.

In the training process of two pianos, the difficulties mainly focus on three aspects: The first aspect is rhythm; when playing two pianos, the rhythm of the two must be strictly consistent; for piano duet, neat rhythm is the premise; if even neat rhythm cannot be achieved, it will be very terrible. The second aspect is that, in terms of intensity, the intensity changes in performance are integrated into the mood changes in music, which are closely combined with music language, tempo, and breath. Piano is a multipart instrument, and the performance of two pianos is more abundant in the voice part, so it is very important to change and contrast the dynamics between the part of speech and the part of intonation. Such performance will only give people a noisy feeling without any aesthetic feeling of music. The third aspect is that, in terms of timbre, compared with single piano, two pianos have a richer sense of hierarchy and a fuller symphonic quality [7]. Nevertheless, the timbre of the two pianos should be carried out according to the changes of melody, harmony, voice parts, and tonality, and different timbre should be used to play different voice parts, so as to find the best performance effect.

### 2.2. Research Status of Sound Sequence Recognition

Sequence recognition is a multidisciplinary technology involving signal processing, probability theory, machine learning, musicology, and so on. The following will discuss the research status of sequence recognition from the three directions of speech enhancement technology, robust sequence recognition, and joint sequence enhancement and recognition.

**Figure 1:**
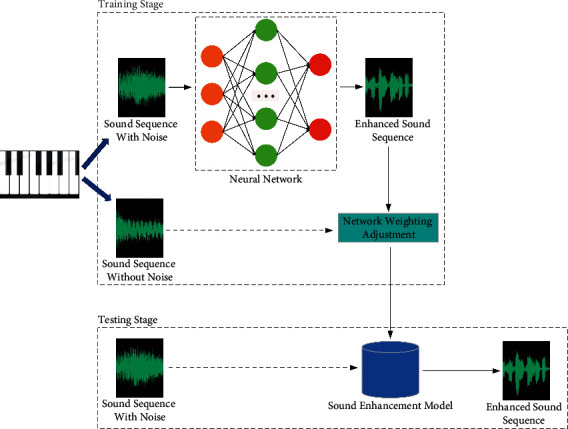
End-to-end sound enhancement model structure diagram.

The goal of speech enhancement is to improve the intelligibility and perceptual quality of noise sequence. Noise reduction model can effectively improve the sound pollution caused by environmental noise or other disturbances. Speech enhancement methods can be divided into classical methods and deep learning methods. Classical methods mainly include spectral subtraction, Wiener filtering, and subspace method. Hinton solved the training difficulties of deep neural networks, making deep learning rise again, and introduced it into the field of speech enhancement recognition. The speech enhancement model with end-to-end structure is shown in Figure 1. These deep learning methods can be divided into end-to-end method, time-frequency masking method, spectrum mapping method, and signal approximation method according to the different audio characteristics and learning objectives processed by neural network [8]. For frequency domain enhancement, the time-frequency domain method only deals with the spectrum representation and still uses the noisy phase in the time domain waveform synthesis stage. In this way, the phase information cannot be truly utilized, which will limit the further improvement of the algorithm performance.

Automatic sequence recognition is a technology that converts sequence signal into text by computer. After this technology was proposed, it has been gradually applied in many fields. From the earliest isolated digital sequence recognition to the present large-scale sequence recognition, the technology in this field is rapidly innovating and developing in the direction of ease of use and intelligence. However, when these applications are used in real scenes, their performance will be significantly reduced due to the presence of a large amount of environmental noise, which promotes the sound sequence recognition technology to improve its robustness to noise. After the unremitting exploration of this field, researchers have put forward different robust sequence recognition technologies. According to the difference of working mechanism, they can be generally divided into feature domain method and model domain method. By analyzing the intrinsic mechanism of audio signal and noise, scholars put forward the adaptive characteristics of noise, and these characteristics and their normalization are called feature domain method [9]. In feature extraction, artificial neural network and its combination with hidden Markov model were proposed, followed by series system, which combined artificial neural network discriminant feature processing with Gaussian mixture model.

The normalization of the first-order, second-order, and higher-order statistical moments of the feature is realized by the normalization of the cestrum coefficient mean, the cestrum coefficient mean and variance, and the histogram equalization, so as to facilitate the better application of the model to the feature. The model domain method can be further divided into general adaptation and specific noise adaptation by modifying the acoustic model parameters to consider the impact of noise [10]. General adaptive methods convert acoustic model parameters to compensate for the mismatch between training and testing conditions. These methods are universal and applicable not only to noise compensation but also to other types of acoustic changes. The model adaptation can be carried out by supervised mode and unsupervised mode [11]. In supervised mode, the correct text can be referred to to guide the model to evolve in the correct direction. In the unsupervised mode, the decoding process is usually divided into two steps: first decoding the utterance using the initial model to generate a virtual decoding result, and then obtain the final decoding result by combining the adaptive process. A simple and effective model adaptation method is multicondition training, that is, using different noise and different SNR data under different conditions to train the model, so that the model contains the knowledge of possible interference; this method can achieve good performance in predictable environment.

### 2.3. Advantages of Neural Network Model for Sound Sequence Recognition

With the rapid development and updating of neural network model, the recognition technology of speech sequence has gradually become a teaching method to improve the quality of teaching with its powerful function and simple humanized interface management. Diversified sequence software is also playing an increasingly important role and has a big effect in practical piano teaching. First of all, the imitation teaching mode of “copying a copy of a gourd” was no longer adopted in the past. The use of neural network-based intelligent teaching software in the classroom can help students truly achieve the teaching effect of synchronizing “sight” and “sound,” realizing a comprehensive perception of the music score and making the music present a three-dimensional image in the classroom. Secondly, the music is limited not only to stay in the listening stage but also to solve the technical problems of the performer and cannot directly understand the sound effect of the music score [12]. Software to record music also has the original paper score convert MIDI audio and video files, and it can also be based on the speed of the work, the level of touch key, and the different tone to make careful editing processing; further expression of emotion is beneficial to the cultivation of the students of music and can strengthen the rhythm of the training.

According to the difficulties encountered in the two-piano teaching and training in Section 2.1, the advantages of the intelligent sequencer based on the neural network model in the two-piano teaching are explained: The first is the rhythm control of the sequencing software in the training of two pianos; using the staff window tool in the software, it can show the two main parts of the two pianos; clicking play, it will play the music in the form of melody rhythm, so that the player has a deeper understanding of the overall rhythm of the work. The second is using sequence software; Adding a few rhythm-controlling notes for different styles can give the performer a clearer understanding of the rhythm of the piece, not only furthering the performer's understanding of the piece, but also helping the performer develop a more efficient playing behavior [13]. The third is the dynamics control of the sequence software in the training of two pianos. In the multipart works of two pianos, how to deal with the dynamics comparison between the parts is more important. In order to better grasp the level of the voice part, the performer has to analyze and mark on the paper score. The fourth is timbre processing of sequence software in two-piano training if there is no association and design of sound in the player's brain; relying on the traditional teaching mode cannot achieve good results. The sound source on the software helps players to improve the association and design of timbre in music works.

## 3. Algorithm Design

### 3.1. Preprocessing of Sound Sequence

The sequence signal can be regarded as stationary signal in a short time frame (10 ms∼30 ms), and the statistical characteristics of the time spectrum of the sequence and noise are often very different in the time-frequency domain. Therefore, in both the traditional signal processing method and the currently widely studied deep learning method, usually, the sequence signal with noise is transformed into the frequency domain by STFT and then demised, enhanced, or detected.

**Figure 2:**
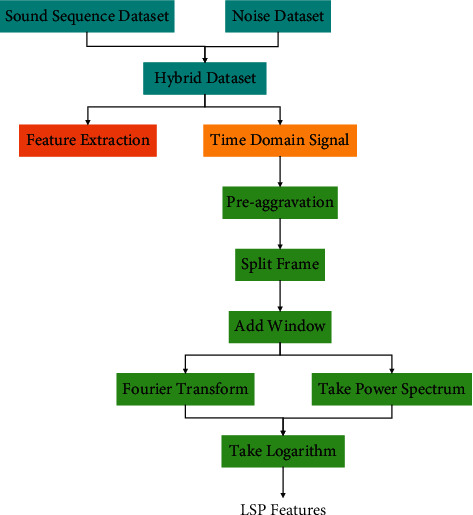
LSP features extraction process.

Compared with raw data, constructed features are more robust for model learning. In the early studies of speech enhancement based on deep learning, the selection of features such as MFCC, GFCC, and MRCG can improve the performance of the system to varying degrees. However, in recent years, with the increase of training data volume and model complexity, the potential of deep learning model to mine better features than manual design has been gradually explored [14]. Researchers prefer to feed rawer data into deep learning models that automatically learn features. LPS is a time-frequency domain feature that is widely used at present. It only takes the dynamic range of logarithmic suppressed sequence and otherwise does no processing on the time-frequency domain amplitude spectrum, reserving more original information. The process for extracting LSP features from mixed data is shown in Figure 2.

Vocal detection refers to the task of detecting whether there is an instrument playing from the sound signal. It is widely used in sequence signal processing. In general, vocal music detection is the most important task in the preprocessing of sequence signal. Therefore, the algorithm is often required to have low latency and strong noise robustness. At present, there are mainly methods based on threshold decision criteria, statistical model, and deep learning. The threshold decision criterion method is the most classical algorithm, which usually needs to extract features with strong discrimination ability and strong noise robustness from sound signals. However, when the signal-to-noise ratio decreases, the feature space is no longer linearly separable, and the performance will decline sharply. Machine learning-based methods transform sound signals into clustering or classification problems, and their models have high complexity, but their noise robustness is often better [15]. In recent years, more and more researchers have begun to apply deep learning to detection tasks. With the powerful data-driven ability of supervised deep learning, they have achieved good results. However, at present, vocal music detection under low SNR and unmatched noise is still a very challenging task.

Vocal music detection based on deep learning can be regarded as a dichotomous problem. Whether there is a training target is marked on the sequence time frame. A label value of 1 means there is an instrument playing, and a label value of 0 means there is no instrument playing. For dichotomous problems, binary cross entropy is often used as the loss function, which is defined as follows:

(1) $$\begin{array}{c} L=-\sum_{t=1}^{T}\left[{\bar{Z}}_{t}\ln\;{\hat{Z}}_{t}+\left(1-{\bar{Z}}_{t}\right)\ln\left(1-\hat{Z}\right)\right], \end{array}$$

where ${\bar{Z}}_{t}$ and $\hat{Z}$ represent the ideal label and estimated value of the test, respectively. The performance of deep learning-based vocal music detection under unmatched noise is often unsatisfactory because of the insufficient generalization ability of common models. By sharing parameters, multitask learning technology can make multiple tasks have the same expression at the level of abstraction, which is equivalent to imposing soft constraints on parameters, which means that the generalization ability of tasks will be improved. Voice enhancement and vocal music detection have a strong correlation, which can be regarded as the probability of estimating the presence of sound order in time-frequency domain and time domain, respectively. Li proposed adding speech enhancement to the vocal detection model to form a multitask learning framework, forcing the model to better understand the phonetic order in the shared part, so as to improve its generalization ability.

In addition, in recent years, deep learning has been widely used in various tasks of sequence signal processing, but the current deep learning models are often complicated and have many parameters, which makes their deployment difficult. Even if you can reduce the size of the network by distilling knowledge, you have to deploy multiple models at the same time to perform multiple tasks on the same terminal, which still puts a lot of strain on the hardware. Speech enhancement is only used as an aid to improve generalization ability during training, and the layer of speech enhancement will be removed in the prediction stage of the model [16]. Therefore, we try to balance the two tasks and merge them into one model in a hard-shared mode, so that it can complete the two tasks simultaneously and in parallel with less computation, which will be of great significance for the deployment of the model.

**Figure 3:**
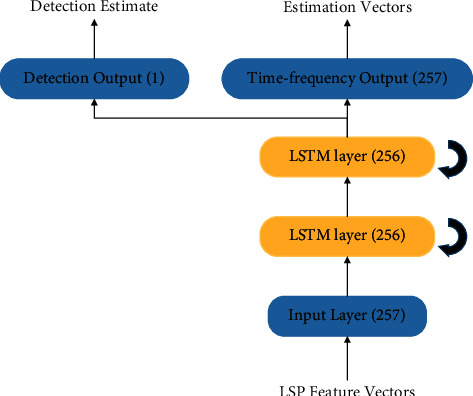
Multitask learning sound enhancement and detection model.

Figure 3 shows the structure of the multitask learning model proposed in this paper. The model adopts the parameter sharing mode of hard sharing. By sharing the bottom two LSTM layers (512 units in each layer), the two tasks can extract some common features together [17]. Due to the strong correlation between the two tasks, the model directly takes the two fully connected output layers as the private modules of the two tasks, which can greatly reduce the number of parameters in the whole network.

For the training of multitask model, there are many loss functions of the model, which can be integrated and optimized simultaneously by adopting the form of hyperparameter *α* weighted sum. *L*_EV_ represents the estimated vector loss function, and *L*_DE_ represents the estimated loss function of detection. The loss function of the multitask model *L*_MT_ can be defined as

(2) $$\begin{array}{c} L_{\text{MT}}=L_{\text{EV}}+\alpha L_{\text{DE}}. \end{array}$$

### 3.2. Sound Sequence Recognition Model

Aiming at the problem of robust noise recognition, the algorithm is studied from many aspects: (1) Simple CBRD modules are stacked, and the end-to-end acoustic model structure is designed with time connectionism algorithm to simplify the model complexity. (2) For the situation of the same sound and different words, the language model was introduced to further modify the transcription results of the phonological sequence model recognition. (3) The combination scheme of front-end enhancement model and back-end recognition model is studied, and the influence of different combination methods on recognition effect is explored.

**Figure 4:**
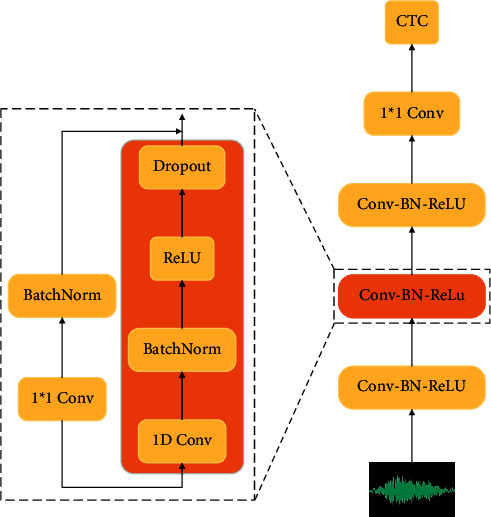
The overall structure of the sound recognition model.

In order to simplify the design process and refer to the cutting-edge work of sequence recognition in recent years, this paper considers designing a single neural network as the main structure of the sequence recognition model [18]. The model in this chapter uses stacked one-dimensional convolution to replace the acoustic model for sequence recognition. Considering the feasibility of actual training duration, the model only includes convolution, BatchNorm, ReLU, and Dropout operations, which can further accelerate the GPU training process and increase the iteration speed after reducing the complexity. The loss function used in the training process is temporal connectionism, which avoids the alignment problem between audio signals and transcribed text in the recognition process. The model uses Mayer filter to calculate the spectrum, in which the window length is 20 ms, the frame shift is 10 ms, and the probability distribution of characters is predicted frame by frame. The whole model structure is composed of stacked residual blocks, and all blocks have the same output dimension. The framework structure of the proposed model is shown in Figure 4. The main feature extraction part of the model consists of several residual blocks, and each residual block is composed of several CBRD units. Each unit consists of only 1D convolutional neural network, BatchNorm, ReLU activation function, and Dropout. In front of the residual block, there is a layer of CBRD unit which is mainly used for the transformation of the convolutional network [19]. After the residual block in *M* layer, another layer of CBRD unit is added with the convolution of 1 × 1, which is mainly used for channel transformation and postprocessing. Finally, the CTC is trained as the loss function.

The model parameters are shown in Table 1. The input and output of each block are connected by residual connections, which are first convolved by 1 × 1 to ensure that the input and output have the same dimensions, and then optimized by adding a BatchNorm layer, and the final output is made by activation functions and Dropout. This subblock design can improve GPU training speed to some extent. Each subblock can be fused into a GPU core. Dropout is not used in the verification stage, and BatchNorm can be fused with the previous convolution.

For the convenience of processing, the audio signal is segmented into frames when it is input into the neural network for training. Normally, alignment is carried out. However, for the recognition of sound sequence, due to the different speed of sound, this alignment will cost a lot of manpower and material resources to construct the dataset, while the chain timing classification can be used for end-to-end training, without the need for orderly pairing between input and output [20]. The similarity between the input signal and the output tone sequence can be measured by CTC without known conditions. Thus, the correlation between the sequence signal and the corresponding text is described. When CTC is used as loss function, the similarity between input sequence and output sequence is calculated, and the CTC function layer is connected to the output end of neural network to calculate the similarity between input sequence and output sequence.

Languages are abstracted symbols that have evolved to form relatively fixed forms of expression. The grammatical structure serves as a standard for people's language use, and the language model can be used to analyze the structural information of various symbols in it. The model can be built on the basis of the preceding and following relationships of character occurrences and can be used to determine whether the sentence structure conforms to the specifications set by the model.

N-gram model is simple in structure and easy to understand and has applications in many fields. It is based on Markova hypothesis, under which the probability of the occurrence of the NTH word in a sentence is only related to the historical *n* − 1 word and has nothing to do with the earlier historical information. According to statistics, the probability of a group phrase forming a sentence is related to each word, and they are connected by probability [21]. The more words that make up a sentence, the more accurate it will be, but the calculation process will also become more complex and increase the amount of computation. Its accuracy is enhanced by complexity, that is, by increasing *N*. To effectively prevent the occurrence of abnormal probability items, additive smoothing operation can be added:

(3) $$\begin{array}{c} r^{*}=\left(r+1\right)\frac{n_{r+1}}{n_{r}}. \end{array}$$

In the above formula, the result of repeated *n*-element *r* model is written as *n*_*r*_, and *r*^*∗*^ is the total number of *n*-gram occurrences. Compared with the simple addition operation, this method adds the amount of calculation, but this amount of computation is acceptable for modern computers, and in combination with embedded systems, real-time computation for text processing can be achieved. Kneser-Ney smoothing algorithm adopts the strategy of interval filling, and its combinative calculation plays different roles. The Kneser-Ney smoothing algorithm is the best among the three smoothing algorithms and is widely used in *n*-gram model.

**Figure 5:**
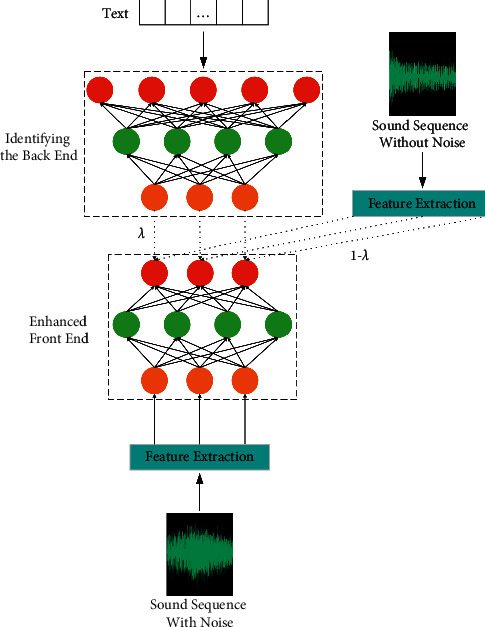
Schematic diagram of joint training of enhanced recognition model.

Based on the investigation and summary of relevant research progress at home and abroad, combined with academic frontier methods, this paper focuses on exploring the combination of front-end enhancement and back-end recognition. Combining with the status quo at home and abroad, this paper puts forward strengthening the joint training model and identification model, using back-propagation neural network to identify the error part back to the enhancement of neural network to enhance the part of the network weights; plan of sampling is added in the training process, reducing the spread model and speeding up the convergence.  Figure 5 shows a schematic diagram of the joint training of the enhancement recognition model. In this scheme, the enhancement front-end and the back-end recognition are organically combined for joint training [22]. The method of training and enhancing the front-end and recognizing the back-end will often reduce the coupling degree between the two modules, and the enhancement process will bring unexpected loss of sequence information, which may reduce the accuracy of recognition. By means of joint training, the two modules are fused into a whole, reducing the differences between the two modules and forming information complementarity between the enhanced front-end and the back-end recognition.

On the existing recognition algorithms, we find that combining different structural front-end models enhances the processing of noisy sequences. Similarly, the joint training process of recognition models can be accelerated by using sampling strategies, either in end-to-end or non-end-to-end form, to eventually output feature vectors. In the task of fusing the enhanced front-end and identifying the back-end, the input features from the enhanced front-end or clean sound order are selected according to a certain probability distribution. At the initial stage of training, because the performance of the enhanced model is not improved, the features of the input back-end recognition may not be able to better represent the audio information, leading to difficulties in model convergence [23]. Using the feature of clean sequence can correct the model, reduce the divergence of the model, and speed up the convergence. Parameters *λ* are added to control the proportion of clean features and enhanced model output features with a certain probability to prevent overcorrection.

## 4. Experiment and Analysis

### 4.1. Evaluation Indicators

In this experiment, two indicators, short-term objective intelligibility (STOI) and perception estimation of speech quality (PESQ), were used to evaluate the speech enhancement effect of the model. They, respectively, evaluated the two main factors of sequence perception: sequence quality and intelligibility. STOI is obtained by measuring the correlation of the short-term envelope between clean and enhanced sequences, with a value ranging from 0 to 1, also usually expressed as a percentage. PESQ uses auditory transformations to generate loudness spectra and compares the loudness spectra of clean and enhanced sequences to produce scores corresponding to predicted MOS scores, which range from −0.5 to 4.5.

In order to verify the results of the proposed multitask speech enhancement and detection model (LSTM-MTL), the following representative single-task models were selected as the baseline and comparison methods: The first is DNN-SE: a deep neural network consisting of four fully connected hidden layers, with each layer containing 1024 hidden units. The second is LSTM-SE: it is composed of two LSTM layers and a fully connected output layer. Each LSTM layer has 512 units and the output is 257 dimensions of time-frequency masking. The third is Sohn: this is one of the most classical speech order recognition algorithms based on statistical model, and the likelihood ratio of speech activity is taken as the output in this experiment. The fourth is LSTM-VAD: it consists of two LSTM layers and a fully connected output layer. The LSTM layer has 512 and 256 units, respectively, and the output is 1-dimensional VAD information.

### 4.2. Experimental Results

#### 4.2.1. Experimental Results of Pretreatment

**Figure 6:**
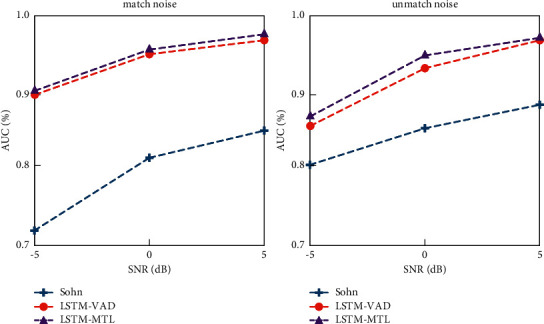
AUC results at *p* = 60%.

Figure 6 shows the AUC comparison results of LSTM-MTL (*p* = 60%) and VAD baseline models under various SNR matched and unmatched noises. LSTM-VAD and LSTM-MTL based on deep learning are far better than the classical Sohn method under matching noise. Under the noise of 5 dB, the AUC of both deep learning models can reach more than 95% regardless of matched noise or unmatched noise. However, in the unmatched noise test, the performance of the model based on deep learning will be seriously affected by the reduction of SNR. However, the AUC of LSTM-MTL is better than that of LSTM-VAD under low SNR thanks to the improvement of generalization ability brought by the multitask learning structure of LSTM-MTL.

As shown in Table 2, under babble noise with low SNR, the performance of LSTM-VAD deteriorates seriously. This is because, at 0 dB and −5 dB, the target sequence has the same or even lower energy as the background sound in babble noise, so it is difficult for the model to determine whether the target sequence exists. However, as LSTM-MTL added speech enhancement task learning, its ability to judge the existence of the target sequence under low signal-to-noise ratio babble noise was improved. Finally, compared with LSTM-VAD, AUC improved 2.8% under −5 dB and 2.4% under 0 dB.

**Figure 7:**
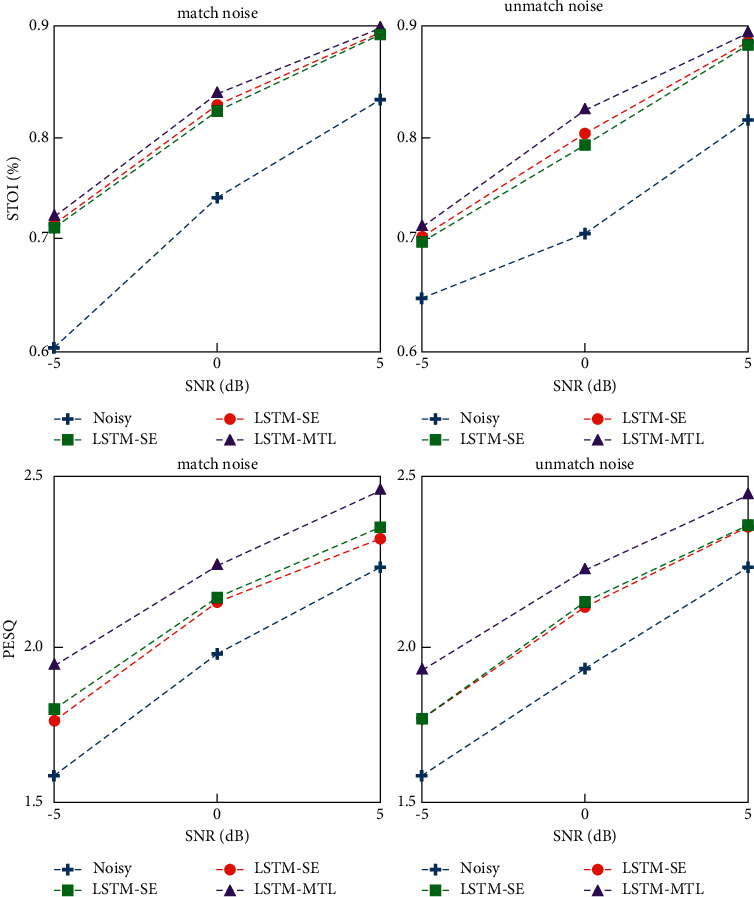
PESQ and STOI results at *p* = 60%.

Figure 7 compares the speech enhancement results of LSTM-MTL (*p* = 60%) with other methods. It can be seen that the results of LSTM-MTL and LSTM-SE are better than those of DNN-SE, which also proves that the stronger timing modeling ability of LSTM network can indeed bring gains to the speech enhancement results. The enhancement effect of LSTM-MTL was between LSTM-SE and DNN-SE. Although the difference was small, the results of PESQ and STOI of LSTM-MTL were always slightly worse than those of LSTM-SE. This is because the training dataset is filled with zero segments, which makes the proportion of sequence segments lower and ultimately leads to the loss of speech enhancement ability. To investigate this problem, we used three training sets *p* = 60%, *p* = 70%, and no fill (*p* = 78%) to train LSTM-MTL and tested it in the test set *p* = 60%. It is worth noting that PESQ and STOI have weak perception of nonsequenced segments, and the filled zero segment has almost no change in the final enhancement evaluation result. However, *p* = 60% can make the proportion of sequenced and nonsequenced segments relatively balanced, making the evaluation result of AUC more accurate. Therefore, we choose to test all tests in *p* = 60%.

**Figure 8:**
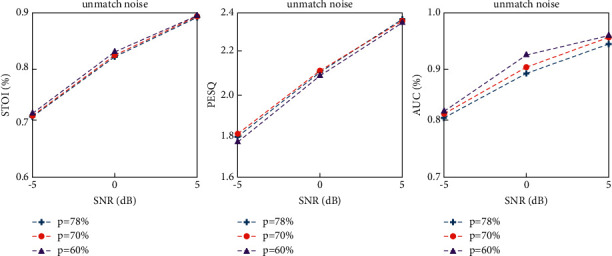
PESQ, STOI, and AUC results with different sound sequence occupancy ratios.

As shown in Figure 8, the less the training set is filled, the better the PESQ and STOI results of speech are, but the overall change is not obvious. For VAD tasks, the more the training set is filled, the more balanced the proportion of speech is and the better the performance of VAD is. Therefore, the training model with *p* = 60% training set can not only achieve good voice enhancement effect but also obtain the optimal VAD effect.

#### 4.2.2. Experimental Results of Sound Sequence Recognition

Since the target of speech enhancement and sequence recognition are different, there will be a certain degree of mismatch during the first recognition after sequence enhancement, and the role of the speech enhancement model is to weaken the interference of noisy sequences to the recognition, and at the same time to mark some key sounds. In our method, we use noisy sequences to train the speech enhancement model. The neural network can make adaptive adjustment based on the existing weight and find the adaptive point between enhancement and recognition. In the scheme, the front-end of the enhancement is the aforementioned three typical neural network enhancement models: AET, R-CED, and UNetSMB. The back-end recognition was pretrained on LibriSpeech, and the effect was improved by combining with the sequence model. In order to control variables, our speech enhancement models are trained on a uniform dataset. In the process of robust recognition, we test the recognition results under different noise and different SNR conditions.

Table 3 shows the performance of the robust sequence recognition model at a high SNR. It can be seen from the table that, under the condition of a high SNR, the model combining speech enhancement and recognition can still improve the recognition effect with noise sequence to a certain extent and achieve a lower word error rate, in order to more intuitively represent the performance improvement of each combination model.

Table 4 shows the robustness of the sequence recognition model under low SNR. From the table, we can see that the model trained jointly under low SNR conditions has a certain degree of improvement in recognition accuracy, but due to the severe noise pollution under low SNR conditions, it still has a high error rate even when the denoising module is added.

On the whole, using the fusion enhancement and recognition model can increase the coupling degree between the front-end enhancement model and the back-end recognition model and has a lower error rate than the enhancement before recognition. Enhanced integration of the front-end and the back-end recognition will result in a lower error rate than directly cascading models. But, even so, we see that, overall, there is still a big gap between the recognition effect of noisy sequence and that of clean sequence. On the basis of the existing model, the performance and generalization of the model can be improved by adding the sequence and noise datasets.

#### 4.2.3. Experimental Results of Application Comparison

Finally, the sequence recognition model is used for auxiliary analysis of two-piano teaching, and an automatic solution is proposed around the sequence correction, rhythm correction, and strength correction, which lays a foundation for exploring the practical application of neural network model in piano teaching. Sequence preprocessing is an important functional module of the system, which is used to remove the noise in the sequence and enhance the sequence part. Then, the phonetic order is recognized. At present, most of the existing sequence recognition models and application products are provided to users in the way of integration of existing technologies. Their common pain point is that the product has poor antinoise ability, and the recognition accuracy may deteriorate due to the influence of environmental noise in practical application. In this paper, the algorithm mentioned above is applied to this particular field and some adaptive improvements are made in the face of the fact that the recognition of the sequence is always disturbed. After the training, we compared the model with the traditional method, and the experimental results are shown in Table 5.

By comparison with the traditional method, we find that although the recognition model can more accurately identify the player's wrong sequence, compared with the machine correction, the artificial correction shows a better effect, although the traditional method has a lower preparation rate of error recognition. This does not prevent the feasibility of our proposed method, which will be further studied in the future from the two aspects of performance improvement and application.

## 5. Conclusions

Based on the gradual intervention and development of neural network model in the teaching of two pianos, this paper analyzes the problems of rhythm, strength, and timbre in the training of two pianos from the characteristics and difficulties of two-piano performance and deeply studies the function and advantage of the recognition of sound sequence. In order to efficiently preprocess the front-end sequence, a multitask online real-time model was proposed for simultaneous sequence enhancement and detection in the same model. We explore the combination of the enhancement model and the recognition model for the recognition of voice sequence under the condition of noise. Considering the reasons such as large number of parameters and cumbersome recognition process in current sequence recognition models, this paper proposes a sequence recognition model with stacked multilayer CBRD modules, which can directly recognize audio signals without cumbersome signal processing methods. Through the cascade of trained enhancement model and recognition model, the two models can be better integrated and adapted to alleviate the incompatibility between enhancement and recognition model caused by different tasks. Experimental results show that our method can effectively improve the recognition accuracy of the sequence recognition model under noise conditions. At the same time, the comparison of the traditional manual and sequence recognition training methods for two pianos proves that our method is equally effective in improving the training efficiency and performance quality of players. In addition, the sequence recognition also meets the application needs of special industries, and the technology can be extended to other specific areas of speech recognition applications with special grammar rules. Aiming at the generalization shortcomings of general speech recognition, taking the sequence recognition as a breakthrough, customized speech recognition schemes for different specific fields are developed.

## Figures and Tables

**Table 1:** Model parameters.

Module name	Number	Number of submodules	Output channels	Dropout
InputConv	1	0	256	0.2
CBRD1	2	4	256	0.2
CBRD2	2	4	512	0.2
CBRD3	2	4	512	0.3
OutConv	1	0	256	0.3
1 *∗* 1 Conv	1	0	1	0

**Table 2:** Comparison of AUC results under nonmatching noise.

Input noise type	−5 dB			0 dB			5 dB		
	F2	Ba	Avg.	F2	Ba	Avg.	F2	Ba	Avg.
Sohn	82.6	76.2	78.5	90.4	82.7	85.1	91.8	89.3	90.3
LSTM-VAD	93.4	70.8	83.6	97.2	86.3	90.8	97.6	94.9	96.5
LSTM-MTL	95.9	78.3	86.4	98.3	87.5	93.2	98.5	97.2	97.6

**Table 3:** High signal-to-noise ratio model identification results.

Input noise type	Whitt (%)	F16 (%)	HF channel (%)
Noisy	18.6	14.8	17.8
AET	16.2	12.6	15.3
R-CED	13.5	10.5	12.8
UNetSMB	12.7	9.3	12.1

**Table 4:** Low signal-to-noise ratio model identification results.

Input noise type	Whitt (%)	F16 (%)	HF channel (%)
Noisy	64.2	36.7	52.4
AET	53.7	31.2	44.5
R-CED	48.5	25.4	38.7
UNetSMB	43.6	20.8	32.6

**Table 5:** Comparison of traditional and sound sequence recognition methods applied.

Training methods	Traditional method (%)	Identification model (%)
Error recognition rate	87.6	94.3
Correction rate	93.2	90.8
Accuracy rate after training	95.4	92.7

## Data Availability

The datasets used in this paper are available from the corresponding author upon request.
